# A multifunctional MRI model based on IVIM and DKI predicts HIF-1α, Ki-67, and VEGF status in breast carcinoma

**DOI:** 10.3389/fonc.2025.1652932

**Published:** 2025-10-20

**Authors:** Zhengtong Wang, Fan Zhao, Laimin Zhu, Ning Mao, Weiwei Wang, Wenwen Zhao, Hao Yu, Yunxi Li, Chongchong Li, Xiuzheng Yue, Yueqin Chen, Zhanguo Sun

**Affiliations:** ^1^ Department of Medical Imaging, Affiliated Hospital of Jining Medical University, Jining, China; ^2^ Department of Breast Surgery, Affiliated Hospital of Jining Medical University, Jining, China; ^3^ Department of Radiology, Yantai Yuhuangding Hospital, Qingdao University, Yantai, China; ^4^ Department of Cardiology, Affiliated Hospital of Jining Medical University, Jining, China; ^5^ Philips Healthcare, Beijing, China

**Keywords:** diffusion-weighted imaging (DWI), diffusion kurtosis imaging (DKI), intravoxel incoherent motion (IVIM), hypoxia-inducible factor-1 alpha (HIF-1α), Ki-67, vascular endothelial growth factor (VEGF), apparent diffusion coefficient (ADC), true diffusion coefficient (D)

## Abstract

**Purpose:**

The research aims to explore the predictive significance of diffusion-weighted imaging (DWI), diffusion kurtosis imaging (DKI), intravoxel incoherent motion (IVIM), and their integrated models in relation to Hypoxia-inducible factor-1 alpha (HIF-1α), Ki-67, and vascular endothelial growth factor (VEGF) expression levels in breast carcinoma.

**Materials and Methods:**

This retrospective study included 104 patients with pathologically confirmed breast carcinoma from our institution as the training set, while an external validation cohort of 91 eligible patients was recruited from another tertiary medical center. Two independently working radiologists analyzed IVIM-derived parameters apparent diffusion coefficient (ADC), true diffusion coefficient (D), perfusion-related diffusion coefficient (D*), and perfusion fraction (f), and DKI-derived parameters mean diffusivity (MD) and mean kurtosis (MK). Receiver operating characteristic (ROC) curves were constructed for evaluation of diagnostic efficacy. The outcomes of the multivariate logistic regression model were employed to create a nomogram of the combined model for molecular marker status prediction.

**Results:**

High expression levels of HIF-1α, VEGF, and Ki-67 were consistently associated with lower D, MD, and ADC values, and higher perfusion-related D*, f, and MK values (all P<0.05). ROC curve analysis showed that among the individual parameters, the D value exhibited the highest predictive efficacy (Area Under the Curve, AUC = 0.724). A D value ≤ 0.88×10^-3^ mm^2^/s should strongly suggest high HIF-1α expression. ROC curve analysis revealed that the f parameter was the most powerful single indicator for predicting VEGF expression (AUC = 0.882). In clinical practice, an f value ≥ 29.82% can serve as a key imaging biomarker suggesting high VEGF expression, i.e., active tumor angiogenesis. ROC curve analysis indicated MD as the most predictive single parameter for Ki-67 expression (AUC = 0.762), showing significantly greater efficacy than D* (Z = 2.022, P = 0.043). Thus, an MD value ≤ 2.21×10^-3^ mm^2^/s strongly suggests high tumor proliferative activity. In the training set, the combined models integrating select parameters from IVIM and DKI showed significantly higher predictive performance (AUCs: 0.852-0.923) compared to individual parameters. This performance was replicated in the external validation set (AUCs: 0.841-0.918), with no statistically significant difference in AUCs between the training and external validation sets according to DeLong’s test (all P > 0.05). Moreover, the solid line provided a better approximation of the ideal dotted line, indicating higher predictive accuracy of the nomograms (P = 0.59, 0.40, and 0.08). According to the decision curve analysis (DCA), the predictive model provided a substantial net clinical benefit.

**Conclusion:**

Our findings suggest that IVIM may be usefully combined with DKI to help predict the expression levels of Ki-67, HIF-1α, and VEGF in breast cancer, generating hypotheses for future research. Furthermore, the diagnostic efficiency of the parameters D* and f appears to be enhanced by employing more low b-values (<100–200 s/mm²). These results require confirmation in prospective, multi-center studies.

## Introduction

Breast carcinoma is currently recognized as a leading malignancy and primary contributor to cancer-related mortality in women. Moreover, recent trends indicate a growing incidence of breast cancer diagnoses among younger individuals (1). From a molecular viewpoint, breast carcinoma is a highly heterogeneous tumor type, posing considerable challenges for the accurate evaluation and implementation of personalized medicine into clinical practice (2). Factors such as tumor hypoxia, neovascularization, and proliferation contribute to the development of breast cancer heterogeneity, which has pivotal roles in the treatment response, prognosis, and recurrence of breast cancer (3, 4). Biomarkers including HIF-1α, Ki-67, and VEGF are recognized indicators of intratumoral heterogeneity, reflecting adaptive responses to hypoxia, proliferative activity, and angiogenic potential, respectively (5–7). Although biopsy remains the gold standard for diagnosis, its invasive nature and limited sampling constrain its ability to fully capture spatial and tumor heterogeneity. Thus, non-invasive imaging techniques capable of characterizing the tumor microenvironment comprehensively are increasingly needed.

In an era of personalized medicine, breast imaging becomes an essential component for breast cancer in terms of diagnosis, staging, treatment, and surveillance. Magnetic Resonance Imaging (MRI) is valuable in tumor disease assessment due to its freedom from ionizing radiation, high soft tissue resolution, multi-sequence imaging capability, and high repeatability properties (8). With the gradual refinement of tumor diagnosis and treatment, the quantitative evaluation of tumor imaging has become increasingly important over the years (9). The DWI’s ADC can reflect the water molecules’ random movement (Brownian motion). However, the movement of water molecules within tissues may not be accurately represented by the ADC value due to the influence of blood microcirculation and tumor variability (10). Upon application of the double-exponential diffusion attenuation model, the IVIM can exhibit water molecules regarding tissue diffusion as well as perfusion parameters without using a contrast medium thus compensating for the limitations of traditional DWI (11). In addition, DKI can effectively account for the intricacy of tissue microstructure while providing diffusivity and kurtosis data, thereby yielding more accurate diffusion information (12). Therefore, multifunctional magnetic resonance imaging based on diffusion-weighted imaging provides a more accurate reflection of tumor heterogeneity.

Although DWI, IVIM, and DKI have been increasingly applied in predicting molecular markers across malignancies such as non-small cell lung cancer, hepatocellular carcinoma, endometrial carcinoma and breast carcinoma (13–17), few studies have systematically evaluated and compared the diagnostic performance of these techniques-both individually and in combination-for predicting HIF-1α, Ki-67, and VEGF expression in breast cancer. Moreover, the potential of integrated models remain underexplored. Therefore, this study seeks to quantitatively assess and compare the value of DWI, IVIM, DKI, and combined models in predicting the expression levels of these biomarkers.

## Materials and methods

### Subjects and clinical factors

**Figure 1:**
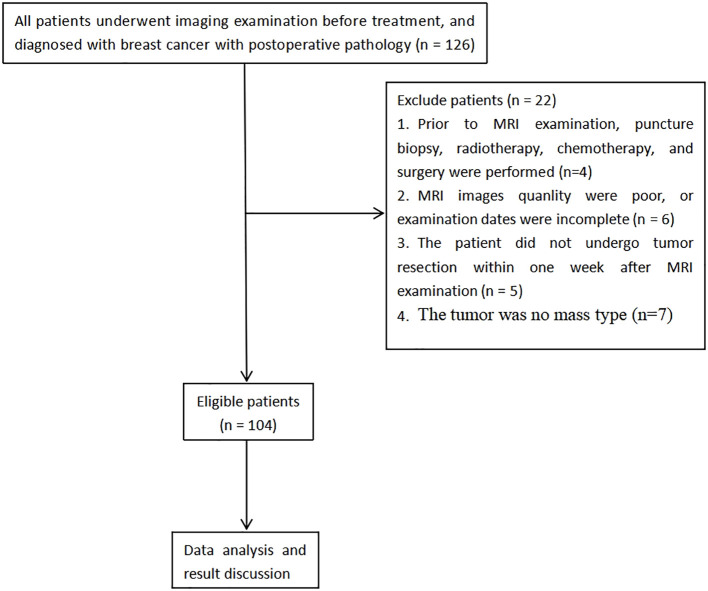
Flowchart of analysis of enrolled patients.

A total of 126 patients diagnosed with breast carcinoma, confirmed through pathological examination from January 2021 to December 2023, were recruited retrospectively. The adopted inclusion criteria involved: a) Subjects were all examined via conventional MRI, IVIM, and DKI, b) no contraindications for MRI examination, and c) complete clinicopathological data were obtained. The following exclusion criteria were implemented: a) patients who had undergone any prior treatment including core needle biopsy, radiotherapy, chemotherapy, or surgery before MRI examination, b) cases where MRI image quality was poor (defined as images with severe motion artifacts or insufficient signal-to-noise ratio for reliable ROI placement) or examination dates were incomplete, c) patients that had not undergone tumor resection within one week following MRI, and d) cases where the tumor did not present as a mass type. Ultimately, 104 patients who met the specified inclusion and exclusion parameters were enrolled for study (**Figure 1**). Additionally, 91 breast carcinoma patients were concurrently enrolled from another tertiary medical center, applying the same inclusion and exclusion criteria, to form an external validation cohort for assessing the model’s generalizability.

### MRI technique

A GE Discovery 750W 3.0T MR scanner was utilized, featuring a dedicated 8-channel phased-array coil designed to encompass both breasts of all patients in a prone position. The MRI sequences included DKI, fast spin-echo T1-weighted imaging (FSE-T1WI), fat-suppressed fast-recovery fast spin-echo T2-weighted imaging (FRFSE-T2WI), DWI, and IVIM. The parameters employed to acquire FSE-T1WI images were as follows: echo time (TE)=10 ms, repetition time (TR)=420 ms, number of excitations (NEX)=1, and matrix = 320×288. The following sequences were employed to obtain FRFSE-T2WI images: TR = 6000 ms, TE = 88 ms, NEX = 2, matrix = 320×288. The DWI parameters were NEX = 2, TR = 3600 ms, matrix=320×288, TE = 73 ms, b-values=0, and 1000 s/mm^2^. The IVIM parameters were defined as follows: TE = 90 ms, thirteen b-values of 0, 20, 30, 50, 70, 100, 150, 200, 500, 700, 1000, 1500, 2000 s/mm^2^, matrix=128×128, NEX increased from 1 to 6, and TR = 2500 ms. DKI was acquired using the following sequence: NEX = 2, matrix=128×128, b-values at 0, 1000, 2000 s/mm^2^. TE = 90 ms, field of view (FOV)=35 cm×35 mm, TR = 5000 ms, slice thickness=4 mm, and slice spacing= 0.4mm. The above sequence layers are all slice thickness=4mm, FOV = 35 cm×35 mm, and slice spacing=0.4mm.

### MRI evaluation

Processing of resulting data on DWI, IVIM, and DKI was completed by virtue of the Function tool MADC and DKI software (GE Healthcare, USA) to acquire parametric maps.

For DWI, ADC maps were generated using a mono-exponential model: S_b_/S_0_ = exp (-b·ADC), where b denotes the diffusion factor, specifically, b=0 s/mm^2^ for one volume and b=1000 s/mm^2^ for the other (18).

The fitting equation reported previously was employed to calculate IVIM-derived parameters: S_b_/S_0_ = (1 - f) × exp (-b × D) + f × exp[-b × (D* + D)], where S_b_ represents the intensity of the MRI signal possessing diffusion weighting b, S_0_ the intensity of non-diffusion-weighted signal, and ADC the apparent diffusion coefficient. D signifies the true diffusion coefficient for water molecules concerning the pure diffusion. The pseudo-diffusion coefficient D* corresponds to incoherent microcirculation related to perfusion and the microvascular volume fraction is denoted by the perfusion fraction f (18).

The parameters derived from DKI were obtained using the equation: S_b_ = S_0_·exp(-b^2^·D^2^ + b·D^2^·K/6), where S_b_ and S_0_ correspond to signal intensities at different b-values (0 s/mm^2^ and others, respectively). MD denotes the diffusion coefficient under normal conditions, adjusted for the non-Gaussian effects, while MK denotes non-Gaussian diffusion behavior (18).

Two radiologists (ZW with 5 years of experience together with WW having 8-year experience in breast MRI) who were blinded to all pathological results and clinical data, drew the ROIs independently. Since IVIM and DKI images displayed a low signal-to-noise ratio, the tumor’s most enormous layers were selected to determine the ROI as per the DWI and T2WI images in the axial direction (b = 1000 s/mm^2^). Three ROIs, each with 50~150 mm2 area, were drawn on the tumor’s solid part, excluding the regions of cystic degeneration, bleeding, and necrosis as much as possible, and then copied the ROI onto the D, D *, f, MD, and MK pseudocolor images. The average values of the quantitative parameters from both radiologists were calculated for final analysis. In cases of significant discrepancy in ROI placement (defined as a > 10% difference in measured value for any parameter), the images were jointly re-reviewed to reach a consensus. This approach, which focused on solid components, was chosen to minimize the confounding effects of necrosis and hemorrhage on parameter calculation, even though it may not fully capture the entire tumor’s heterogeneity.

### Pathological assessments

All participating patients underwent surgical interventions, specifically, radical mastectomy, modified radical mastectomy, and breast-conserving surgery in 68, 24, and 12 cases, respectively. The clinical database was utilized to extract multiple pathologic characteristics, such as histologic type, tumor size, ER/PR/HER-2/HIF-1α/VEGF/Ki-67 status, histologic grade, tumor subtype, and patient age. All immunohistochemical evaluations of HIF-1α, Ki-67, and VEGF were performed on postoperative surgical specimens obtained from the resected tumor mass. All surgically resected specimens were carried out by two independent pathologists (with working experience of 6 and 15 years), depending on levels of either estrogen receptor (ER), ki-67, human epidermal growth factor-2 (Her-2), as well as progesterone receptor (PR). Positivity for PR and ER was determined in cases where 10% or more nuclei exhibited positive staining. Ki-67 expression ≥14% was considered positive, while levels below this threshold were considered negative. Her-2 staining intensity was semiquantitatively scored 0 points for negative, 1+ point for weak, 2+ points for moderate or 3+ points for strong. The score of 0 or 1+ point meant Her-2 negative, while 3+ score was classified Her-2-positive. For tumors with a score of 2+ points, fluorescence *in situ* hybridization was conducted for further evaluation of gene amplification (19). Four distinct molecular subtypes of breast cancer were identified, namely, Her-2-positive (negative for ER and PR, Her-2-positive), luminal A (Her-2-negative, positive for ER or PR, ki-67≥14%), triple-negative (negative for ER, PR and Her-2), and luminal B (negative for Her-2, positive for ER or PR, Ki-67<14%). Assessment of HIF-1α was conducted by calculating the ratio of cells with positive HIF-1α to the total number of cells and scored as follows: 0 points (0–25%), 1 point (25–50%), 2 points (50–75%), 3 points (75–100%) and 4 points (100%). The intensity of staining was rated on a scale of 0 (no color), 1 (light yellow), 2 (brown), and 3 (dark brown) points. Low expression was defined by staining intensity <2 points alongside cells with positive HIF-1α <2 points. In contrast, a high expression was defined (20). The scoring system was applied to evaluate VEGF immunostaining based on the computed percentage of positive cells (0, no positive cells; 1, ≤25% positive cells; 2, 25%< positive cells ≤50%; 3, >50% positive cells) in addition to staining intensity (0=negative; 1=weak; 2=moderate; 3=high). The grading of these parameters yielded combined scores of 0–2, 3–4, and 5–6 corresponding to negative, positive, and strongly positive results, respectively (21).

### Statistical analysis

MedCalc 19.5.1 (Ostend, Belgium), R version 4.0.0 (http://www.r-project.org/), and SPSS 25.0 (IBM Corporation, Armonk, NY, USA) were employed for statistical analyses. The statistical analysis was performed under the supervision of a professional statistician (Juntao Zhang, GE Healthcare PDX GMS medical affairs, Senior Data Scientist). Interclass correlation coefficients (ICCs) were utilized to assess interobserver reproducibility. The criteria adopted for interpretation of ICC agreement were as follows: ICC ≤ 0.20, poor; 0.21<ICC ≤ 0.40, fair; 0.40<ICC ≤ 0.60, moderate; 0.60<ICC ≤ 0.80, good; 0.80<ICC ≤ 1.00, excellent (22). Two quantitative data samples were subjected to the Kolnogorov-Smirnov normality test to evaluate their distribution patterns. Data that followed normal and non-normal distribution were expressed as mean ± standard deviation and median plus interquartile range, respectively. The independent samples t-test was used for normally distributed data, and the Mann-Whitney U test was applied for non-normally distributed data. Different groups were compared using the independent-sample chi-square test. The binary logistic regression analysis was used to identify independent factors. Receiver operating characteristic (ROI) curves were performed to evaluate the diagnostic efficacy of each parameter or model. The Delong test analyzed the values of area under different groups’ ROC curve (AUC). A nomogram was subsequently constructed and validated using decision curve analysis (DCA), together with calibration techniques. The Spearman rank was employed to describe the correlation of each parameter with HIF-1α, VEGF, and Ki-67, respectively. A correlation coefficient (r) of 0.75-1.00 was considered to indicate a good correlation, 0.50-0.74 a moderate correlation, 0.25-0.49 a mild correlation, and 0.24 or lower little or no correlation, p <0.05 denoted a difference of statistical significance (23).

## Results

### Clinical and pathological features

The 104 participants in the study had a mean age of 52.99 ± 9.58 years, with an range from 29 to 71. Within this cohort of 104 cases of breast cancer, 95 (91.3%) were classified as invasive ductal carcinoma and 9 (8.7%) as non-invasive ductal carcinoma. Among the 104 patients, 11 (11%) were categorized as luminal A subtype, 19 (18%) as HER-2-positive subtype, 51 (49%) as luminal B subtype, and 23 (22%) as triple-negative subtype (**Table 1**). In the training set, no statistically significant differences were found in tumor genotypes between the high- and low-expression groups of HIF-1α, VEGF, and Ki-67 (P = 0.42, 0.24, and 0.55, respectively). This lack of significant association was consistently observed in the external validation cohort, with corresponding P values of 0.22, 0.14, and 0.22.

**Table 1:** Clinical and pathological features of enrolled patients.

Feature	Date
Age (years), mean+SD	52.99 ± 9.58
Mean tumor size (cm), mean+SD	2.56 ± 1.09
Histologic grade, n (%)	
1	7 (6.7%)
2	44 (42.3%)
3	53 (51.0%)
Histologic type, n (%)	
Invasive ductal carcinoma	95 (91.3%)
Non-invasive ductal carcinoma	9 (8.7%)
ER, n (%)	
Negative (-)	42 (40%)
Positive (+)	62 (60%)
PR, n (%)	
Negative (-)	53 (51%)
Positive (+)	51 (49%)
HER-2, n (%)	
Negative (-)	51 (49%)
Positive (+)	53 (51%)
HIF-1α	
High	65 (62%)
Low	39 (38%)
VEGF	
High	64 (61%)
Low	40 (39%)
ki-67	
<14%	42 (40%)
>14%	62 (60%)
Tumor subtype, n (%)	
Luminal A	11 (11%)
Luminal B	51 (49%)
HER-2-positive	19 (18%)
Triple-negative	23 (22%)

HIF-1α: Hypoxia inducible factor-alpha; ER: estrogen receptor; VEGF: vascular endothelial growth factor; PR: progesterone receptor.

### Interobserver agreement

The two radiologists reported ICCs of 0.887 [95% CI: 0.837–0.922], 0.917 [95% CI: 0.880–0.943], 0.860 [95% CI: 0.800–0.903], 0.880 [95% CI: 0.828–0.917], 0.909 [95% CI: 0.868–0.937], and 0.863 [95% CI: 0.805–0.905] for ADC, D, D*, f, MD, and MK, respectively. These results reflect a significant level of agreement between observers.

### Diagnostic efficiency of DKI, DWI, and IVIM-derived parameters for HIF-1α expression

**Figure 2:**
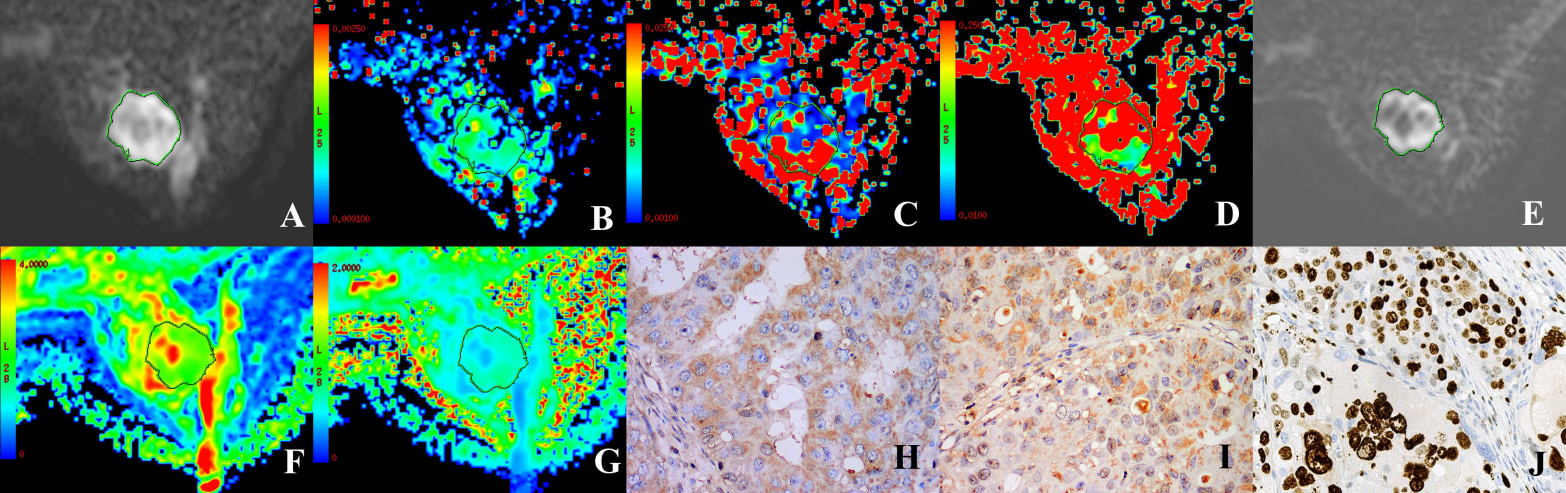
A 37 year-old female patient with HER-2-positive cancer of the right breast. **(A, E)** Grayscale map (IVIM, DKI) presenting a b-value of 1000 s/mm^2^ was selected to determine the ROI. **(B–D)** Maps in pseudo-colors for D, D*, and **(F)** D = 0.8 × 10^-3^ mm^2^/s, D*=71.49 × 10^-3^ mm^2^/s, f = 39.29%. **(F, G)** Maps in pseudo-colors for MD and MK. MD = 1.85 × 10-10^-3^ mm^2^/s, MK = 0.84. **(H–J)** Immunohistochemical staining images for HIF-1α (high expression), VEGF (strongly positive), and Ki-67 (positive) in cases of invasive ductal carcinoma of the breast.

**Figure 3:**
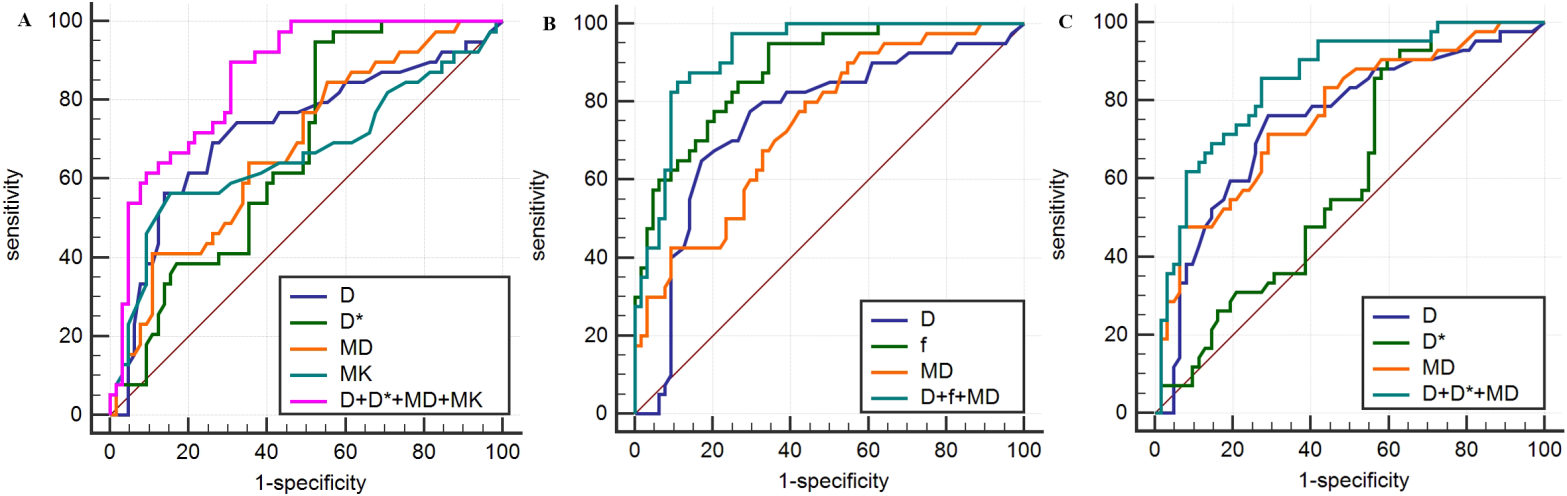
Analysis of parameters derived from IVIM and DKI for prediction of HIF-1α **(A)**, VEGF **(B)** and Ki-67 **(C)** expression in breast carcinoma based on ROC curves.

In the training set, the D and MD values were significantly lower in tumors in the high-expression than the low-expression HIF-1α group (p < 0.05). Additionally, the high-expression group was observed with increased values of D*, f, and MK compared with the low-expression group (p < 0.05). D [odds ratio(OR) = 0.001, P <0.001], D* (OR = 1.805, P = 0.018), MD (OR = 0.168, P = 0.011), and MK (OR = 2034.665, P = 0.015) as significant predictive factors of HIF-1α. ROC curve analysis showed that among the individual parameters, the D value exhibited the highest predictive efficacy (AUC = 0.724). A D value ≤ 0.88×10^-3^ mm^2^/s should strongly suggest high HIF-1α expression. The AUC of the combined model incorporating MD, D, MK, and D* was 0.860 with an accuracy of 77.9%, representing a modest but statistically significant improvement (Z=2.878~3.881, all P<0.05). A similar trend was observed in the external validation set. The combined model demonstrated comparable performance in the external validation set, achieving an AUC of 0.841, which was not significantly different from that in the training set (DeLong’s test, Z = 0.262, P = 0.794). This indicates that the combined model provides a more reliable non-invasive assessment of tumor hypoxia (**Tables 2**, **3**; **Figures 2**, **3**).

**Table 2:** Performance of the model combining IVIM and DKI in determination of the expression status of HIF-1α.

Parameter	Training (n = 104)			External validation (n = 91)		
	HIF-1α		P-value	HIF-1α		P-value
	High	Low		High	Low	
ADC (×10^-3^mm^2^/s)	0.95 ± 0.09	0.97 ± 0.10	0.26	0.94 ± 0.09	0.96 ± 0.08	0.41
D (×10^-3^mm^2^/s)	0.82 ± 0.15	0.93 ± 0.16	0.001	0.81 ± 0.12	0.90 ± 013	0.001
D* (×10^-3^mm^2^/s)	26.29 (20.16, 47.28)	23.53 (18.52, 27.44)	0.003*	28.66 (15.23, 58.78)	26.42 (12.45, 41.35)	0.02*
f (%)	31.73 (26.68, 36.36)	26.63 (21.73, 29.82)	0.007*	31.84 (16.37,45.15)	26.26 (18.54,46.89)	0.002*
MD (×10^-3^mm^2^/s)	2.16 ± 0.45	2.47 ± 0.52	0.002	2.08 ± 0.40	2.35 ± 0.50	0.005
MK	0.78 ± 0.11	0.71 ± 0.14	0.006	0.75 ± 0.09	0.70 ± 0.13	0.03

Date are presented as mean ± SD for normally distributed parameters (ADC, D, and MD) and median (IQR) for non-normally distributed parameters (D*, f, and MK); HIF-1α, Hypoxia inducible factor-alpha; MD, mean diffusion; ADC, apparent diffusion coefficient; MK, mean kurtosis; *Mann-Whitney U test

**Table 3:** Performance of the model combining IVIM and DKI in determination of the expression status of HIF-1α, VEGF and ki-67.

Parameters	Training (n = 104)						External validation (n = 91)					
	AUC	95%CI	Cutoff	Sensitivity (%)	Specificity (%)	Accuracy (%)	AUC	95%CI	Cutoff	Sensitivity (%)	Specificity (%)	Accuracy (%)
HIF-1α												
D	0.724	0.627-0.807	0.88	69.2	73.8	70.2	0.704	0.599-0.795	0.90	57.14	76.79	61.5
D*	0.674	0.575-0.763	32.73	94.9	47.7	59.6	0.652	0.545-0.749	37.55	97.14	33.93	64.8
MD	0.680	0.582-0.768	2.69	41.0	89.2	71.2	0.668	0.562-0.764	2.57	42.86	87.50	69.2
MK	0.665	0.565-0.754	0.68	56.4	84.6	70.2	0.644	0.537-0.742	0.68	48.57	80.36	68.1
Combined model	0.860	0.779-0.921	–	89.7	69.2	77.9	0.841	0.750-0.910	–	77.14	80.36	76.9
VEGF												
D	0.752	0.658-0.832	0.86	77.5	70.3	71.2	0.731	0.628-0.819	0.83	80.00	60.71	61.5
f	0.882	0.804-0.937	29.82	95.0	65.6	78.8	0.869	0.782-0.931	27.18	77.14	80.36	78.0
MD	0.744	0.649-0.824	2.03	80.0	56.3	66.3	0.701	0.596-0.793	1.98	74.29	62.50	67.0
Combined model	0.923	0.854-0.966	–	85.0	89.1	87.5	0.918	0.841-0.965	–	82.86	89.29	83.5
ki-67												
D	0.748	0.653-0.828	0.86	76.2	71.0	71.2	0.748	0.647-0.834	0.83	78.38	61.11	69.2
D*	0.607	0.506-0.701	35.71	90.5	40.3	54.8	0.611	0.503-0.712	37.55	94.59	33.33	60.4
MD	0.762	0.668-0.840	2.21	71.4	71.0	70.2	0.721	0.617-0.810	1.98	75.68	64.81	68.1
Combined model	0.852	0.769-0.914	–	85.7	72.6	77.9	0.847	0.757-0.914	–	81.08	75.93	75.8

Cut-off values were determined using Yonden’s index; HIF-1α, Hypoxia inducible factor-alpha; CI, confidence interval; MD, mean diffusion; MK, mean kurtosis; AUC, area under the ROC curve; VEGF, vascular endothelial growth factor.

### Diagnostic efficiency of DWI, IVIM, and DKI-derived parameters for VEGF expression

In the training set, comparative analyses revealed that relative to the low-expression group, the high-expression VEGF group had lower ADC, D, and MD values, along with higher D*, MK, and f values (P<0.05). Furthermore, D (OR = 1.805, p = 0.018), f (OR = 1.805, p = 0.018), and MD (OR = 1.805, p = 0.018) served as independent predictors for assessing VEGF expression status. ROC curve analysis revealed that the f parameter was the most powerful single indicator for predicting VEGF expression (AUC = 0.882). The parameter f exhibited a significantly larger AUC than D and MD (Z = 2.036, P = 0.042; Z = 2.375, P = 0.018). In clinical practice, an f value ≥ 29.82% can serve as a key imaging biomarker suggesting high VEGF expression, i.e., active tumor angiogenesis. The combined model that incorporated D, f, and MD produced an AUC of 0.923, achieving an accuracy of 87.5%, demonstrating improved performance compared to the use of D or MD alone (Z = 3.357, P<0.001; Z = 3.927, P<0.001). Notably, however, the combined model did not significantly outperform f alone in terms of AUC (Z = 1.837, P = 0.066). A similar trend was observed in the external validation set. The generalizability of the combined model was further supported by the external validation results, which showed a consistently high AUC of 0.918. No statistically significant difference was found compared to the training set performance (DeLong’s test, Z = 0.089, P = 0.929). This suggests that the f parameter may play a central role in assessing angiogenesis (**Tables 3**, **4**; **Figures 2**, **3**).

**Table 4:** Performance of the model combining IVIM and DKI in determination of the expression status of VEGF.

Parameter	Training (n = 104)			External validation (n = 91)		
	VEGF		P-value	VEGF		P-value
	High	Low		High	Low	
ADC (×10^-3^mm^2^/s)	0.94 ± 0.09	0.98 ± 0.09	0.019	0.92 ± 0.08	0.98 ± 0.08	0.002
D (×10^-3^mm^2^/s)	0.82 ± 0.16	0.94 ± 0.14	<0.001	0.81 ± 0.13	0.91 ± 0.12	<0.001
D* (×10^-3^mm^2^/s)	27.01 (21.41, 46.54)	20.30 (18.19, 27.43)	<0.001*	29.40 (15.23, 58.78)	25.03 (12.45, 36.98)	0.009*
f (%)	32.30 (28.73, 38.15)	23.75 (19.78, 27.45)	<0.001*	32.52 (21.59,46.89)	23.59 (16.37,33.52)	<0.001*
MD (×10^-3^mm^2^/s)	2.11 ± 0.39	2.54 ± 0.54	<0.001	2.05 ± 0.39	2.39 ± 0.49	<0.001
MK	0.77 ± 0.12	0.72 ± 0.13	0.038	0.75 ± 0.10	0.71 ± 0.12	0.14

Date are presented as mean ± SD for normally distributed parameters (ADC, D, and MD) and median (IQR) for non-normally distributed parameters (D*, f, and MK); MD, mean diffusion; ADC, apparent diffusion coefficient; VEGF, vascular endothelial growth factor; MK, mean kurtosis; *Mann-Whitney U test

### Diagnostic efficiency of DWI, IVIM, and DKI-derived parameters for Ki-67 expression

In the training set, the high-expression Ki-67 group exhibited significantly decreased ADC, D, and MD values, along with increased D* and f values, compared to the low-expression group (P < 0.05). The parameters D (OR = 0.007, P = 0.002), D* (OR = 1.080, p = 0.005), and MD (OR = 0.120, p = 0.002) emerged as predictors that could independently assess the status of Ki-67 expression. ROC curve analysis indicated MD as the most predictive single parameter for Ki-67 expression (AUC = 0.762), showing significantly greater efficacy than D* (Z = 2.022, P = 0.043). Thus, an MD value ≤ 2.21×10^-3^ mm^2^/s strongly suggests high tumor proliferative activity. Furthermore, the combined model of D, D*, and MD achieved an AUC of 0.852, with an accuracy of 77.9%, which was superior to that of the D, D*, and MD parameters (Z=2.368~4.199, all P<0.05). A similar trend was observed in the external validation set. However, no statistically significant difference was found for the D*parameter. No statistically significant difference was observed in the AUC values between the training set and the external validation set (AUC = 0.847) as assessed by DeLong’s test (Z = 0.073, P = 0.942). This demonstrates the model’s strong and generalizable predictive performance. This result indicates that integrating diffusion and perfusion information provides a more comprehensive perspective for the non-invasive assessment of tumor proliferation (**Tables 3**, **5**; **Figures 2**, **3**).

**Table 5:** Performance of the model combining IVIM and DKI in determination of the expression status of Ki-67.

Parameter	Training (n = 104)			External validation (n = 91)		
	Ki-67		P-value	Ki-67		P-value
	High	Low		High	Low	
ADC (×10^-3^mm^2^/s)	0.94 ± 0.10	0.98 ± 0.10	0.047	0.93 ± 0.09	0.97 ± 0.09	0.036
D (×10^-3^mm^2^/s)	0.82 ± 0.16	0.94 ± 0.14	<0.001	0.80 ± 0.12	0.91 ± 0.13	<0.001
D* (×10^-3^mm^2^/s)	33.71 ± 16.41	25.69 ± 8.20	0.004	27.83 (15.23,58.78)	25.46 (12.45,45.89)	0.072*
f (%)	31.78 (26.93, 36.82)	26.60 (21.73, 29.77)	0.002*	31.65 (16.37,45.15)	26.45 (18.54,46.89)	0.004*
MD (×10^-3^mm^2^/s)	2.10 ± 0.40	2.54 ± 0.51	<0.001	2.03 ± 0.37	2.40 ± 0.49	<0.001
MK	0.77 ± 0.11	0.73 ± 0.14	0.072	0.74 ± 0.09	0.71 ± 0.13	0.109

Date are presented as mean ± SD for normally distributed parameters (ADC, D, and MD) and median (IQR) for non-normally distributed parameters (D*, f, and MK); MD, mean diffusion; ADC, apparent diffusion coefficient; MK, mean kurtosis; *Mann-Whitney U test

### Predictive nomogram assessing the efficiency of parameters derived from DWI, IVIM, and DKI in determination of HIF-1α, VEGF, and Ki-67 expression

**Figure 4:**
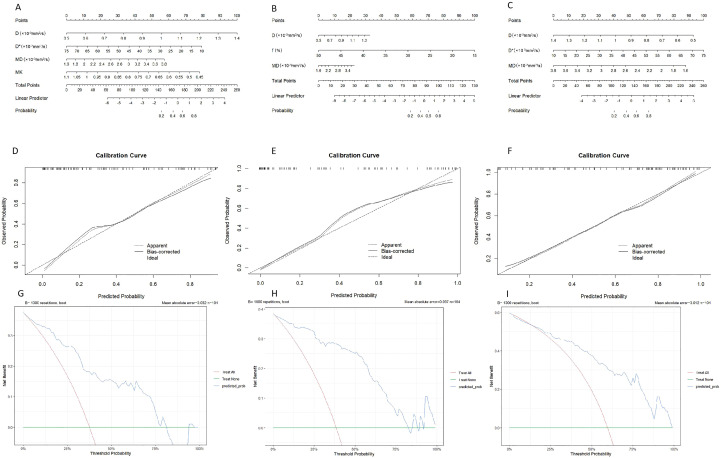
Nomogram for predicting the expression patterns of HIF-1α **(A)**, VEGF **(B)** and Ki-67 **(C)** in breast carcinoma. Calibration charts for internally validated HIF-1α **(D)**, VEGF **(E)** and Ki-67 **(F)**. **(G–I)** illustrate the results of decision curve analysis (DCA) for the nomogram models.

Based on the final regression analysis, we incorporated independent factors demonstrating significant associations with the diagnostic efficacy of parameters from DWI, IVIM, and DKI for expression of HIF-1α, VEGF, and Ki-67 into multivariate regression analysis, subsequently leading to the development of three nomograms (**Figures 4A–C**). Next, we evaluated the performance of the model by establishing internal calibration curves (**Figures 4D–F**). The probability of HIF-1α, VEGF, and ki-67 expression, as predicted via the nomogram, was set as the X-axis, with the actual probability represented on the Y-axis. The results showed that the ideal dotted line was more approximated by the solid line, suggesting higher predictive accuracy of the nomograms (p = 0.59, 0.40, and 0.08).The decision curve analysis (DCA) described in **Figures 4G–I** highlights the excellent net clinical benefit of the predictive model.

### Correlation analysis

HIF-1α showed mild positive correlations with D*, f, and MK, respectively (r = 0.292, 0.267, 0.276, p<0.05). HIF-1α was mildly correlated with D and MD, respectively (r = -0.375, -0.302, p<0.05). VEGF showed mild and moderate positive correlations with D* and f, respectively (r = 0.390, 0.644, p<0.05). VEGF was mildly correlated with D and MD, respectively (r = -0.425, -0.411, p<0.05). Ki-67 showed mild positive correlations with D* and f (r = 0.242, 0.308, p<0.05). Ki-67 was mildly correlated with D and MD, respectively (r = -0.422, -0.445, p<0.05).

## Discussion

Breast cancer is a multifaceted disease featuring uncontrolled cell growth. The rapid propagation of breast tumor cells is reflected by elevated Ki-67 expression (24). This study demonstrated decreased ADC, D, and MD values in the groups showing high Ki-67 expression relative to those with low expression, in keeping with findings documented in numerous earlier studies (17, 25, 26). The biological mechanism underlying this phenomenon is rooted in the fact that highly proliferative tumor cells result in a substantial increase in cellular density. This increase significantly restricts the diffusion of water molecules, leading to a deviation from Gaussian distribution. According to Meng et al. (17), efficient prediction of high and low Ki-67 expression using the ADC value poses a considerable challenge. This discrepancy may be ascribed to ADC demonstrating the proper diffusion of water molecules together with “false diffusion” caused by the blood microcirculation within capillaries. In this study, the high ki-67 expression group when contrasted with the low expression group exhibited higher values of D* and f, suggesting that the tumor tissue maintains high perfusion levels. However, other investigations have reported no significant correlation between D* and f values and Ki-67 expression (25, 27). Our in-depth analysis suggests that these discrepancies could be closely related to the selection strategy of b-values below 100-200 s/mm² in IVIM sequences, as these low b-values were sensitive to perfusion effects. Research by Zhang et al. (25) demonstrated that a few b-values less than 100–200 s/mm² could reduce the predictive efficiency of parameters D* and f. In this study, the IVIM sequence was selected to set 13 b values, among which 8 were lower than 200 s/mm^2^. The parameters D, D*, and MD were identified as independent predictors of the Ki-67 expression status. Our results further showed that MD had the highest AUC compared with D and D* parameters, which could be attributed to the enhanced ability of DKI to precisely measure the diffusion of water molecules. Additionally, ROC analysis demonstrated that the model combining all three quantitative parameters outperformed the individual parameters. The model demonstrated robust performance in the training cohort and, more importantly, this strong predictive ability was successfully replicated in the external validation set. This finding suggests that an integrated multi-model approach holds promise for enhancing the non-invasive prediction accuracy of Ki-67 expression status in breast cancer. Consequently, it offers a more comprehensive imaging basis for the preoperative assessment of tumor proliferative activity.

Solid tumors commonly exhibit hypoxia, which arises from the rapid proliferation and intense metabolic activity of tumor cells. HIF-1α has been established as the most important regulatory element in the hypoxic responses of tumor cells (5). Here, we observed that compared to the low HIF-1α expression group, the high expression group exhibited lower values of D and MD, along with higher D*, f, and MK values, consistent with previous results (28, 29). We propose the following mechanistic explanations for these findings: (1) HIF-1α promotes glucose uptake by tumor cells, enhancing their proliferation and metabolic ability, leading to enhanced cellular density that restricts water molecule diffusion (30); (2) HIF-1α is known to stimulate tumor angiogenesis by regulating downstream factors such as VEGF, consequently increasing tissue perfusion (31). Notably, the study by Li et al. (28) reported no significant correlation between HIF-1α expression and D*. This discrepancy again underscores the substantial impact of technical protocols in IVIM imaging, particularly the setting of low b-values, on the comparability of research findings. The above study only selected six b values set below 200 s/mm^2^. Data from the present investigation indicate that D, D*, MD and MK acted as factors capable of independently assessing the status of HIF-1α expression. The D (70.2%) had the highest predictive power. Both MD (71.2%) and MK (70.2%) showed superior accuracy, possibly due to the ability of DKI to not only efficiently quantify water molecules for non-Gaussian diffusion features in tissues but also provide more accurate and reliable information regarding tissue microstructure (12). Moreover, the combination of D, D*, MD and MK exhibited greater predictive accuracy compared to each individual parameter. Furthermore, the successful external validation indicates that our model could serve as a reliable tool for non-invasive assessment of tumor hypoxia across different institutions, thereby supporting clinical decision-making. Therefore, utilizing such a combined model can enhance the prediction of HIF-1α, which holds potential implications for estimating tumor sensitivity to radiotherapy or targeted therapies, as hypoxia is often associated with treatment resistance.

VEGF, which facilitates the angiogenic process, serves as a quantitative index for evaluating angiogenesis in tumors (6). In contrast to the VEGF low-expression group, the high-expression group presented elevation of D* and f values, in alignment with previous research findings (15, 32). This variation could be explained by the stimulatory influence of VEGF expression on angiogenesis, thereby enhancing tumor perfusion. Furthermore, relative to the low-expression group, the high-expression group showed lower ADC and D values. This observation may be attributed to the ability of VEGF to induce angiogenesis, thereby supplying the increased nutrient requirement for proliferation, increasing cell density, and further limiting the diffusion of water molecules. In contrast, Yang et al. (15) found that patients with varying VEGF expression levels showed no significant differences in ADC and D values. The reason for this difference may be that VEGF expression results in a higher speed of proton movement in lumens together with a larger number of tumor blood vessels (33). Our study indicated that compared with the VEGF low-expression group, the high-expression group showed a decline in MD values and concomitant increase in MK values, consistent with previous findings (34), possibly because VEGF accelerates cell proliferation and tumor growth, resulting in high tumor heterogeneity and significant deviation of water diffusion from the Gaussian distribution. The values of D, f and MD served as independent variables for assessing the VEGF expression status. Notably, AUC for f was significantly greater than those of the other parameters. Additional ROC analysis indicated that the composite model utilizing the three quantitative parameters provided superior diagnostic performance compared to the individual parameters. Nonetheless, no substantial differences were observed between the f value and the composite model, clearly implying that f serves as the most promising parameter for distinguishing VEGF expression. This has direct clinical relevance, as anti-angiogenic therapies target the VEGF pathway. The IVIM-derived f parameter provides a non-invasive method for assessing tumor perfusion. In the future, it could be used to screen patients for potential benefit from anti-angiogenic therapy or to monitor early treatment response.

Our research has a number of specific limitations that warrant careful consideration. Firstly, the study was conducted on a limited patient cohort, which could potentially introduce bias into the findings. Secondly, the solid components of the tumor were primarily determined as the ROI, rather than encompassing the entire breast cancer, which may not adequately capture the full extent of tumor heterogeneity. Thirdly, we have not yet achieved precise location of MRI and pathology “face to face”. Fourthly, our ROI analysis was restricted to the solid portion of the tumors, and non-mass lesions were excluded. This may introduce selection bias and limit the generalizability of our findings. Fifthly, the precise optimal number of b-values below 100–200 s/mm^2^ for IVIM imaging in breast cancer remains to be determined. Future studies should focus on larger sample sizes, incorporate whole-tumor ROIs to address heterogeneity, improve MRI-pathology alignment, and optimize low b-values for IVIM imaging to enhance the accuracy and clinical applicability of breast cancer characterization. Finally, the external cohort was recruited from a single center with a limited sample size. Future multi-center studies with larger, prospective cohorts are essential to further confirm these findings and facilitate clinical translation.

In summary, the findings from this retrospective study suggest the potential of a multiparametric MRI approach combining IVIM and DKI for non-invasive assessment of tumor biology in breast cancer. Overall, MD appears to serve as a more reliable predictor of Ki-67 expression relative to parameters derived from IVIM, while the f parameter is the most beneficial for distinguishing VEGF expression compared to other functional indicators from IVIM and DKI. The efficacy in assessing HIF-1α expression is enhanced by the D parameter from IVIM, alongside MD and MK from DKI. These hypothesis-generating results highlight a promising direction for future research. In the training set, combined models integrating select parameters from IVIM and DKI showed significantly higher predictive performance (AUCs: 0.852-0.923) compared to individual parameters. While the gain in AUC may be modest, the model’s value lies in its synthesis of complementary biological information-water diffusion, microcirculatory perfusion, and structural complexity-into a more holistic assessment. In addition, the successful external validation of our model demonstrates not only its exceptional generalizability but also its robustness against overfitting. By applying a wider range of b values, specifically those below 100–200 s/mm², the stability and accuracy of D* and f measurements could be significantly improved. Furthermore, our results suggest that utilizing large quantities of b-values below 100–200 s/mm² could enhance the diagnostic efficiency of D* and f parameters.

## Data Availability

The original contributions presented in the study are included in the article/supplementary material. Further inquiries can be directed to the corresponding authors.
